# Medical AI and AI for Medical Sciences: An Editorial

**DOI:** 10.31662/jmaj.2024-0355

**Published:** 2024-12-27

**Authors:** Eiryo Kawakami

**Affiliations:** 1 Department of Artificial Intelligence Medicine, Graduate School of Medicine, Chiba University, Chiba, Japan; 2 Institute for Advanced Academic Research (IAAR), Chiba University, Chiba, Japan; 3 Advanced Data Science Project (ADSP), RIKEN Information R&D and Strategy Headquarters, RIKEN, Kanagawa, Japan

**Keywords:** artificial intelligence, machine learning, deep learning, data-driven research, disease heterogeneity, disease dynamics

In the third wave of artificial intelligence (AI) after 2010, AI and data sciences were rapidly adopted across medical and healthcare fields. Advances in machine learning technologies, including deep learning, have enabled AI to learn directly from large datasets, thereby achieving accuracy similar to, or better than, that of experts on various tasks, such as highly accurate skin cancer diagnosis using skin imaging data ^(1)^ and comprehensive detection of retinal diseases using optical coherence tomography data ^(2)^. Some of these diagnostic technologies have been certified as AI-powered diagnostic devices by US Food and Drug Administration and are implemented in clinical practice. Furthermore, the emergence of generative AI and large language models ^(3)^ improved the relationship between medicine and AI. In the past, AI models were developed from scratch to address specific issues. However, at present, utilizing foundation models that integrate data and specialized knowledge is possible.

Amid rapid technological evolution, individual research methods are changing, and the entire medical research framework is expected to undergo substantial transformation. Modern medical research has followed a hypothesis-driven approach, in which hypotheses are formulated based on biological experiments and tested in large-scale clinical trials. The hypothesis-driven research has established molecular mechanism-based evidence and standardized medical care, which previously relied heavily on physician experience. However, multifactorial diseases such as lifestyle-related diseases, cancer, and inflammatory diseases, which are notable public health issues, are difficult to explain using a few hypotheses. Inevitably, some exceptions do not conform to any hypothesis. Therefore, a research style bypassing basic biological hypotheses as a starting point, as well as collecting and analyzing diverse biological data directly from patients, has emerged; this approach is called data-driven research, which is in contrast to hypothesis-driven research. AI is a major factor of data-driven research, depicting disease characteristics and capturing heterogeneous treatment effects from vast, high-dimensional data.

At present, medical researchers and healthcare professionals are struggling to adapt to the widespread adoption of AI and evolving research frameworks. For humans, omics data, or comprehensive measurements of biomolecules obtained by next-generation sequencing and mass spectrometry, are challenging to interpret in their raw form and require filters, such as applied statistical methods and AI, to derive insights. In clinical practice, AI-powered automated imaging diagnostics and applications that use generative AI are emerging. There is an increasing demand for medical care that fully utilizes these technologies. Even if the development of AI algorithms and data analysis concerns engineers and data scientists, most medical researchers and physicians must have foundational literacy in AI mechanics, such as understanding basic principles, selecting appropriate methods and tools, and recognizing AI limitations.

Sakurada et al. ^(4)^ offered a comprehensive review of AI applications in medicine and research, covering various AI techniques, such as machine learning, deep learning, reinforcement learning, generative models, and large-scale language models, with intuitive explanations. The review is unique in avoiding technical details and explaining concepts in layman’s terms through examples that link concepts to relevant medical issues. This comprehensive overview of AI technology will be useful for medical professionals interested in using AI and for medical researchers exploring the applicability of AI technologies to their research.

The second half of the article by Sakurada et al. ^(4)^ summarizes the historical and current relationships between AI and science and offers insights into future development. One promising direction is the introduction of “patient states” via machine learning. Traditionally, patients have been classified using phenotypic descriptions in natural language; such discrete disease classifications have difficulty in adequately representing heterogeneity within a disease, interrelationships among diseases, and temporal changes in disease status. Using machine learning to represent patients’ conditions based on quantitative data comprehends the interrelations and dynamics of disease concepts.

Another proposed direction involves the introduction of a physics-based framework into biomedical research. Since the 1940s, biomedical research has progressed from a strict elemental reductionism, emphasizing causal explanations rooted in molecular mechanisms. However, life phenomena beyond causal explanations, such as emergence, phase separation, and oscillation synchronization, have been reported, highlighting the need to incorporate a physics-based deductive paradigm into biomedicine. Additionally, machine learning models that incorporates physics principles, such as diffusion model and physics-informed machine learning (PIML) ^(5)^, have been developed and will potentially be used in biomedicine in the future.

A limitation of Sakurada et al.’s ^(4)^ review is the limited presentation of case studies on actual data analysis and clinical applications. Readers interested in incorporating AI into their clinical practice or research benefit from consulting additional reviews and original papers that provide detailed descriptions of specific methods and tools. Furthermore, this review does not specifically address AI applications in drug discovery.

AI and mathematical methods for capturing disease heterogeneity and dynamics remain in their early stages, and efforts to integrate data- and hypothesis-driven approaches are underway. This field offers ample room for exploration, and young researchers and physicians are encouraged to participate.

## Article Information

### Conflicts of Interest

None
